# The Role of Mast Cells in Stroke

**DOI:** 10.3390/cells8050437

**Published:** 2019-05-10

**Authors:** Edoardo Parrella, Vanessa Porrini, Marina Benarese, Marina Pizzi

**Affiliations:** Department of Molecular and Translational Medicine, University of Brescia, Viale Europa 11, 25123 Brescia, Italy; v.porrini@unibs.it (V.P.); marina.benarese@unibs.it (M.B.); marina.pizzi@unibs.it (M.P.)

**Keywords:** mast cells, stroke, neonatal hypoxic-ischemic brain injury, ischemic stroke, brain ischemia, intracerebral hemorrhage, subarachnoid hemorrhage, blood–brain barrier, inflammation

## Abstract

Mast cells (MCs) are densely granulated perivascular resident cells of hematopoietic origin. Through the release of preformed mediators stored in their granules and newly synthesized molecules, they are able to initiate, modulate, and prolong the immune response upon activation. Their presence in the central nervous system (CNS) has been documented for more than a century. Over the years, MCs have been associated with various neuroinflammatory conditions of CNS, including stroke. They can exacerbate CNS damage in models of ischemic and hemorrhagic stroke by amplifying the inflammatory responses and promoting brain–blood barrier disruption, brain edema, extravasation, and hemorrhage. Here, we review the role of these peculiar cells in the pathophysiology of stroke, in both immature and adult brain. Further, we discuss the role of MCs as potential targets for the treatment of stroke and the compounds potentially active as MCs modulators.

## 1. Introduction

Mast cells (MCs) are perivascular resident cells of haemopoietic origin distributed in most tissues surrounding blood vessels, nerves, smooth muscle cells, sebaceous and sweat glands, hair follicles, and synovial membranes [1,2]. MCs are more abundant in the anatomical regions in contact with the external environment, including skin, conjunctiva, nasal mucosa, bronchial airway connective tissue, lung intra-alveolar space, mouth, and subserosal and submucosal layers of the gastrointestinal tract [2,3,4]. Because of their peculiar anatomical location, MCs serve as first immune sentinel cells to respond against invading pathogens and environmental antigens and allergens [1,5,6].

MCs can be found also in the central nervous system (CNS), where their presence has been documented for more than a century [7]. MCs are present in different mammalian brain regions, including meninges, choroid plexus, olfactory bulb, mesencephalon, parenchima of the thalamic and hypothalamic region, hippocampus, and entorhinal cortex [6,8,9,10], where they reside on the abluminal side of the blood vessels [8,9]. Here, MCs are able to communicate with blood vessel cells, neurons, glia, and microglia [8,9]. MCs reach the brain during development, migrating along blood vessels [11]. However, mature MCs are also able to move from the periphery to the brain and their number and distribution can change in response to a variety of physiological and pathological stimuli [8,12,13].

For years, MCs have been mostly studied for their pathogenic role in allergic and anaphylactic responses. However, in the last decades, these cells have gained recognition for their involvement in a number of other physiological and pathological processes [14]. In the CNS, MCs contribute to normal behavioral development and functioning, modulating cognition and emotionality [8,15,16,17]. On the other hand, MCs have been associated to various neuroinflammatory conditions of CNS, including multiple sclerosis, traumatic brain injury, Alzheimer’s disease, Parkinson’s disease, amyotrophic lateral sclerosis, neuropathic pain, migraine, depression, autism spectrum disorder, fibromyalgia syndrome, and finally stroke [6,8,9,18,19,20,21,22,23,24].

Moreover, a link between MC-mediated allergic reactions and cardiovascular (CV) disorders has been recently proposed [25]. An important evidence for the existence of an overlap between allergic and CV disorders comes from the so-called Kounis syndrome, an acute coronary pathology caused by mastocytic activation triggered by allergic reactions [26,27]. Interestingly, brain vascular pathologies, including stroke and cerebral aneurysm (CA), have been described in Hyper-IgE syndrome and DOCK8 deficiency, two genetic disorders characterized by elevated IgE serum levels, recurrent infections, and allergic reactions [28,29,30]. These findings suggest a possible correlation between IgE levels and stroke.

A growing body of evidence indicates a contribution of MCs in pathogenesis of stroke, suggesting that targeting cerebral MCs may provide a feasible neuroprotective strategy against this medical condition. In the present review, we discussed the role of brain MCs in cellular and animal models of stroke, including neonatal hypoxic-ischemic brain injury (NHIBI), ischemic stroke, intracerebral hemorrhage (ICH), and subarachnoid hemorrhage (SAH). Furthermore, we summarized compounds potentially active as MCs modulators in the treatment of stroke.

## 2. MCs Activation

MCs are characterized by the presence in their cytoplasm of hundreds of metachromatic granules containing preformed biologically active mediators. The best studied mechanism of mastocytic activation is that induced by interaction of antigen with its specific IgE antibody linked to FcεRI (high-affinity surface receptors for the Fc region of IgE). However, MCs can be activated by many other physical and chemical stimuli, including trauma, UV light, cold, heat, hypoxia, allergens, cytokines and other inflammatory mediators, complement factors, pathogens and their products, venom components, and endogenous and exogenous peptides [1,3,5,31,32]. 

MCs activation occurs in three phases [6,8,33]. The first and rapid response occurs within seconds after mastocytic activation and consists in the degranulation of MCs, i.e. the release of preformed mediators stored in MCs granules. The main granules contents include histamine, heparin, serotonin, proteases, proteoglycans, cathepsin G, and cytokines such as tumor necrosis factor α (TNF-α). The second phase consists in the rapid synthesis of lipid mediators, including leukotrienes (such as LTB4, LTC4), prostaglandins (PGD2, PGE2), thromboxanes, platelet-activating factor (PAF). Finally, the first two steps are followed by a third phase, the slow release of newly synthetized cytokines occurring within several hours after mastocytic activation. The type of mastocytic activation and the pattern of released molecules depend on the type and strength of the stimuli [34]. 

Interestingly, many of these mediators can induce inflammation, blood–brain barrier (BBB) damage, vasodilatation, and plasma extravasation, and have been associated with stroke [6,8,35,36]. 

## 3. Transcriptional and Epigenetic Regulation of MCs Response

Transcription factors play an important role in MCs development and activation. In particular, transcription factors are critical in modulating the expression of cell-surface receptors and extracellular mediators involved in mastocytic activation [37,38]. For example, nuclear factor of activated T-cells (NFAT), activator protein 1 (AP-1), nuclear factor kappa-light-chain-enhancer of activated B cells (NF-κB) and its signaling component IκB kinase 2 (IKK2), early growth response 1 and 2 (EGR-1, EGR2), and zinc finger E-box–binding homeobox 2 (ZEB2) have been studied for their role in orchestrating the transcriptional response for de novo synthesis of mediators upon mastocytic activation [39,40,41,42,43,44,45]. Moreover, IKK2 has been suggested to participate in the MCs degranulation process [46]. 

The interest for the epigenetic regulation of the mastocytic processes has emerged in the past years [37,38]. Although the epigenetic mechanisms operating in MCs are still largely unknown, recently two epigenetic regulators, tet methylcytosine dioxygenase 2 (TET2) and DNA methyltransferase 3a (DNMT3a), have been shown to regulate MCs functions in rodent models [47,48]. Interestingly, TET2 and DNMT3a mutations are also frequent in patients affected by mastocytosis [49], suggesting a role of these factors in human pathologies. 

Finally, a peculiar epigenetic mechanism in MCs is mediated by the mastocytic protease tryptase. Although MCs tryptase is mainly stored in cytoplasmatic granules, this enzyme has been found also in the nucleus where it catalyzes the clipping of histones H2 and H3, regulating mastocytic differentiation [50,51]. 

## 4. MCs and Stroke

Stroke, the sudden death of brain cells due to lack of oxygen when the blood flow to the brain is lost by blockage or rupture of a vessel, can occur in both immature and adult brain. 

The incidence of perinatal stroke is between 1/2300 and 1/5000 live births, although these values are probably underestimated because the limited data available and complexity of the diagnosis [52,53]. 

Adult stroke is the second leading cause of death and the third cause of disability worldwide [54,55,56]. Moreover, stroke is also a main cause of dementia and depression [57,58]. Overall, about 85% of strokes are ischemic, 10% are due to intracerebral hemorrhage, and 5% are caused by subarachnoid hemorrhage [59,60,61].

**Figure 1:**
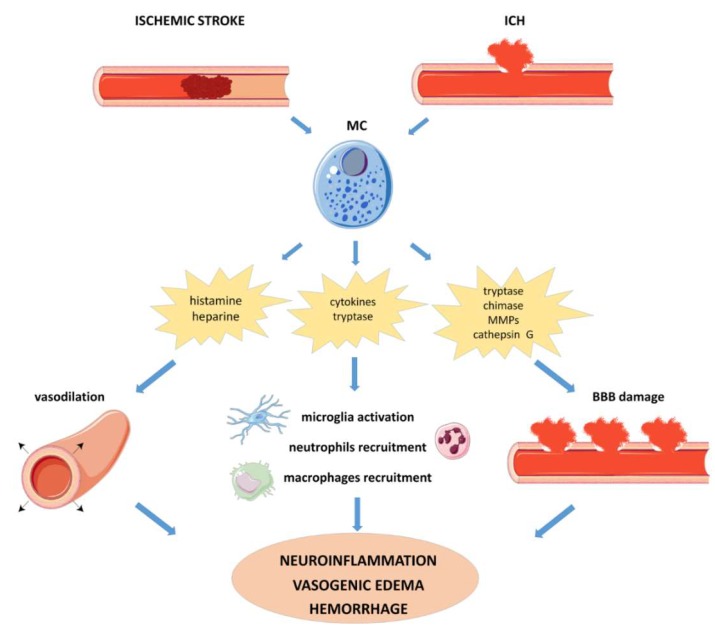
Schematic diagram showing the role of MCs in ischemic stroke and ICH. In the ischemic stroke, after cerebral blood vessel obstruction MCs sense alarm signals from injured parenchymal cells and become activated. In the ICH, the mastocytic activation is mediated by the leakage of blood products from the damaged vessel. Upon activation, MCs release a variety of vasoactive and proinflammatory molecules, including histamine, heparine, cytokines (TNF-α, ILs, chemokines), proteases (tryptase, chymase, MMPs, cathepsine G). The preformed and newly synthetized mediators induce vasodilatation, recruitment of peripheral immune cells toward the infarcted area, and BBB disruption, promoting a sustained neuroinflammation. In ischemic stroke, the pathological scenario supported by MCs activation has been involved in the hemorrhagic conversion mediated by rtPA treatment. In the ICH, the recruitment of inflammatory cells maintain and potentiate the initial BBB leakage, leading to an aggravation of hemorrhage and vasogenic edema. The mechanisms of MCs-mediated pathogenesis of stroke in the adult brain are valid also in the immature brain. BBB: blood–brain barrier; ICH: intracerebral hemorrhage; IL: interleukin; MCs: mast cells; MMP: metalloproteinase; rtPA: recombinant tissue plasminogen activator; TNF: tumor necrosis factor.

**Figure 2:**
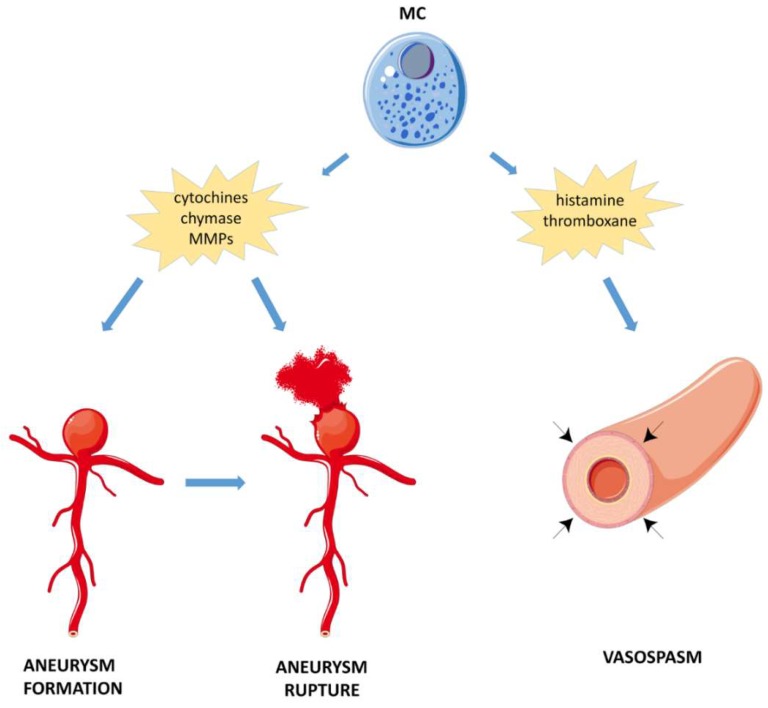
Schematic diagram showing the role of MCs in SAH. MCs have been suggested to play a role in the development of CA and its progression to rupture. Activated MCs infiltrated in the aneurysm site may promote inflammation through the release of mediators including cytokines and proteases (chymase and MMPs). Moreover, MCs can induce expression of MMPs and iNOS in vascular smooth muscle cells, reducing the thinning of CA walls. MCs can also contribute to delayed cerebral vasospasm through the release adenosine-mediated of histamine and thromboxanes. CA: cerebral aneurysm; iNOS: nitric oxide synthase, MCs: mast cells; MMP: metalloproteinase; SAH: subarachnoid hemorrhage.

The role of MCs in the pathogenesis of different subtypes of stroke is discussed in the following paragraphs and in Figure 1, Figure 2, and Table 1.

### 4.1. Ischemia in the Immature Brain

Neonatal hypoxic-ischemic brain injury (NHIBI) is a major cause of acute mortality and chronic neurologic morbidity in infants and children [52,62,63,64]. The pathogenesis of NHIBI is highly complex and involves neuroinflammation, BBB damage, acidosis, growth factor deficiency, and energy failure [65,66,67]. 

Various clues suggest involvement of MCs in the NHIBI pathogenesis [68,69]. MCs-associated genes were upregulated in mice pups subjected to NHIBI [70]. In rat models of NHIBI neuronal injury was linked to a rapid increase of activated MCs and release of TNF-α and histamine in the immature brain [71,72,73], suggesting that mastocytic activation precedes post-injury response of glial, endothelial cells and neurons. In a NHIBI mouse model, the mastocytic growth and differentiation factor interleukin 9 (IL-9) also contributed to brain damage by amplifying activation of MCs [74]. Moreover, the deleterious role of these cells is supported by the effect of MCs stabilizers in inhibiting mastocytic activation and reducing brain damage in rat or mice pups subjected to NHIBI [71,72,74]. 

### 4.2. Ischemia in the Mature Brain

#### 4.2.1. Ischemic Stroke

Ischemic stroke, or brain ischemia, is a global cause of death and disability [59,60]. In ischemic stroke, blood supply to part of the brain is decreased, causing damage to the cerebral tissue surrounding the occluded blood vessel [75]. The ischemic insult triggers a series of pathological processes including excitotoxicity, oxidative damage, apoptosis and inflammation, which eventually leads to cell death [76,77,78]. One of the main pathophysiological features of ischemic stroke is the BBB disruption, an event occurring in almost two-thirds of patients in the first hours from the ischemia onset that causes vasogenic edema, hemorrhagic transformation, and increased mortality [79,80].

Different preclinical studies point out a role of MCs in the pathogenesis of ischemic stroke [81,82]. In a cellular model of ischemic stroke, exposure to oxygen and glucose deprivation (OGD) induced mastocytic activation in a fashion dependent on the anoxic insult duration [83,84,85]. MCs exacerbated neuronal damage in neuron–MC cocultures exposed to OGD [83]. Likewise, the conditioned medium derived from OGD-activated MCs induced neurotoxicity in primary neurons [83]. Interestingly, pharmacological prevention of OGD-induced MCs activation reduced neurotoxicity [83]. The gene expressing the chemokine CCL7, a MCs-derived product reported to be involved in the recruitment of inflammatory cells into the ischemic sites [86], has been found upregulated in the brain of mice subjected to middle cerebral artery occlusion (MCAO) [87]. In a rat model of transient cerebral ischemia, it has been observed a significant increase in the thalamic MCs number and histamine levels after 24 h from the ischemic insult [88]. Experiments on rats that underwent MCAO surgery showed that MCs are early players in the formation of ischemic brain edema [89]. Treatment with the MCs activator compound 48/80 dramatically increased cerebral edema [89]. On the contrary, MC-deficient rats or rats treated with the MCs stabilizer cromoglycate displayed diminished brain swelling, BBB leakage, and neutrophils infiltration [89]. Similarly, BBB breakdown, brain edema, and neutrophils infiltration were attenuated in MC-deficient mice subjected to MCAO or in MCAO wild type mice treated with cromoglycate [90]. Proteomic analysis suggested a role of endoglin, endothelin-1, and metalloproteinase 9 (MMP-9) in the BBB damage promoted by MCs [90]. A proof of the involvement of proteolytic gelatinase enzymes secreted by MCs in BBB damage came from studies on rats subjected to MCAO [91]. After the ischemic insult, activated MCs showed secretion of gelatinase-positive granules that correlated with the brain swelling. Treatment with the compound 48/80 increased gelatinase activity in the ischemic tissue [91], while rats treated with MCs stabilizers or MC-deficient rats presented a reduced global gelatinase-active area [91]. 

Interestingly, the meningeal MCs, rather that parenchimal MCs, appear to be involved in detrimental effect of stroke [92]. In support of this hypothesis, the engraftment of bone marrow-derived cultured MCs into the meninges of MC-deficient mice subjected to MCAO was sufficient to worsen stroke damage [93]. In an immunohistochemical study on brain tissues of patients deceased after ischemic stroke, the authors reported a lack of MCs in the penumbra regions surrounding the necrotic area [94]. These findings further supported the fact that parenchymal MCs may not play a crucial role in stroke.

Treatment options for ischemic stroke are currently very limited, and the only approved pharmacological therapy is the recombinant tissue plasminogen activator (rtPA) [95,96]. Unfortunately, administration of rtPA after 4.5 h from the ischemic event is contraindicated for the risk of hemorrhagic conversion, limiting the use of this drug [97]. Strbian and coworkers studies in ischemic stroke models pointed out a role of MCs in the hemorrhagic conversion promoted by rtPA administration [98]. The treatment of cultured rat MCs with rtPA promoted massive degranulation [98]. In a MCAO rat model, administration of rtPA induced intracerebral hemorrhage formation [98]. Mastocytic stabilization by sodium cromoglycate protected from deleterious effect of rtPA, reducing hemorrhagic conversion, brain swelling, neutrophil infiltration and mortality rate [98]. Similar protective effects were observed in MC-deficient rats [98]. 

#### 4.2.2. Intracerebral Hemorrhage (ICH)

Spontaneous ICH is a severe neurological disorder associated with high rates of mortality and disability [99,100,101,102]. ICH results from the rupture of cerebral blood vessels that causes a rapidly expanding hematoma occurring within brain parenchima. Brain injury after ICH can be divided in two phases. The primary brain injury caused by increased intracranial pressure is followed by a secondary brain injury mediated by the physiological responses to hematoma, such as inflammation [103,104].

Several findings support a role of MCs in ICH [82,105]. In a rat model of intracerebral hemorrhage with autologous blood injection into the basal ganglia, the induction of mastocytic degranulation by treatment with compound 48/80 exacerbated brain damage [106]. Conversely, rats treated with MCs stabilizers or MC-deficient rats showed reduced mortality, brain swelling, hematoma growth and improved neurologic outcome [106]. In another ICH model, mice stereotactically injected into the basal ganglia with collagenase exhibited MCs activation [107,108]. Pharmacological MCs inhibition significantly decreased mortality rate and improved neurologic outcomes by mitigating neuroinflammation and BBB disruption [107,108]. Finally, in a collagenase injection rat model of ICH, pharmacological MCs inhibition counteracted the deleterious effects induced by rtPA administration, such as hematoma growth, hemispheric expansion, mortality, and neurologic dysfunction [109]. 

#### 4.2.3. Subarachnoid Hemorrhage (SAH)

SAH is a severe subtype of stroke characterized by an overall mortality and morbidity of more than 50% [110,111]. SAH is caused in 85% of cases by the rupture of an intracranial aneurysm [111]. Over the past years, a large number of studies reported a role of the inflammatory processes in the pathogenesis of CA [112,113,114].

MCs have been suggested to contribute to vascular diseases including atherosclerosis and aneurysm by triggering inflammation through the release of cytokines and proteinases such as chymase and MMPs [115,116,117,118]. An increased number of infiltrated MCs has been identified in the aneurysm wall in a CA rats model [119]. Similarly, MCs have been found in the aneurysm tissues of CA patients that underwent microsurgical clipping [120,121,122]. Interestingly, MCs expression was markedly increased in ruptured aneurysms [120]. In rats, MCs modulators reduced the size and the thinning of induced CA through inhibition of chronic inflammation [119]. Furthermore, in cocultures of primary rat MCs and smooth muscles cells obtained from intracranial arteries, mastocytic activation induced expression of MMP-2, MMP-9 and inducible nitric oxide synthase (iNOS) in smooth muscles cells [119], revealing a role for MCs in promoting inflammation in CA walls. 

Delayed cerebral vasospasm, the prolonged and intense vasoconstriction of arteries in the subarachnoid space after CA rupture, has been recognized as an important cause of poor outcome in SAH [123]. MCs have been reported to be associated with vasospasm because of their increased number found in the artery walls adjacent to aneurysm of patients who died after SAH [124]. Adenosine and inosine have been proposed as responsible for the vasoconstrictive effect mediated by MCs activation [125]. The MCs-induced vasoconstrictive effect was significantly decreased by treatment with a combination of histamine and thromboxane inhibitors [125].

## 5. MCs Modulation: A Promising Strategy in Stroke Treatment

Over the years, various strategies targeting intracellular and extracellular MCs mediators have been developed, mainly to treat allergic disorders [37]. Some of these MCs modulators have been demonstrated as effective therapeutic agents against stroke in preclinical studies [24]. The MCs modulators active in stroke models are discussed below and in Table 2.

PEA (palmitoylethanolamide) is an endogenous lipid amide distributed in different mammalian tissues, especially the brain [126]. Several reports indicate that PEA can reduce MCs activation in a variety of cellular and in vivo experimental models [127,128,129,130,131,132,133]. Thanks to its anti-inflammatory and analgesic properties, PEA has been investigated for the treatment of several pathologies, including stroke [134]. The molecule is active in protecting against ischemic damage in cellular and animal models of brain ischemia [135,136] and NHIBI [136]. In particular, in rats subjected to MCAO, PEA decreased the release of MCs derived chymase and tryptase [137]. 

Luteolin is a flavonoid that exhibit anti-inflammatory, antioxidant, neuroprotective and anti-carcinogenic activities [138,139]. Luteolin and its congeners have been reported to have a potential for the treatment of several pathologies, including brain ischemia [140,141,142,143] and NHIBI [144]. Moreover, in line with studies showing that luteolin is able to reduce mastocytic activation [145,146,147], the molecule prevented OGD-induced MCs degranulation and reduced neurotoxicity promoted by OGD-activated MCs [83].

Notably, recent findings indicate the combination of PEA and luteolin synergistically reduces MCs-mediated neurotoxicity and neurons susceptibility to hypoxic stress in a cellular model of brain ischemia [83]. Moreover, the association between PEA and luteolin is effective in decreasing the ischemia-induced MCs infiltration and expression of chymase and tryptase in a rat model of ischemic stroke [148].

Sodium cromoglycate (also referred as cromolyn) is an FDA-approved MCs stabilizer used to prevent symptoms associated with asthma [149]. Several studies point out a neuroprotective role of this MCs modulator in preclinical stroke models. Cromoglycate is effective in limiting brain damage in NHIBI, preventing MCs migration, and lowering glial activation and brain atrophy [71,72,74]. Pharmacological MCs inhibition by cromoglycate reduced ischemic brain swelling, perivascular gelatinase activity, BBB leakage, and neutrophil accumulation in rats and mice subjected to ischemic stroke [89,90,91]. Moreover, cromoglycate treatment inhibited hematoma growth and decreased neurological deficits and mortality in rat model of hemorrhagic stroke [106]. Finally, cromoglycate administration was able to reverse adverse effects of rtPA treatment in both ischemic stroke and ICH rat models [98,109], suggesting a potential use of this MCs stabilizer in combination with thrombolytic agents.

Intravenous immunoglobulin (IVIG) is an FDA-approved drug used to treat various inflammatory and autoimmune diseases. IVIG contains mainly IgG and is obtained from the blood of healthy donors [150]. In models of brain ischemia or ICH, IVIG treatment was shown to attenuate BBB damage, brain edema, infarct area as well as production of proinflammatory cytokines [108,151,152,153,154]. Interestingly, it has been reported that IVIG could activate the mastocytic inhibitory receptor FcγRIIB [155]. In the ICH collagenase mouse model, IVIG activated FcγRIIB/SHIP1 pathway, inhibiting calcium mobilization and stabilizing MCs [108].

Hydrogen (H_2_) gas inhalation is recognized as a therapeutic and preventive antioxidant intervention able to reduce the levels of strong oxidants such as hydroxyl radicals and peroxynitrite [156]. H_2_ gas inhalation therapy was neuroprotective in preclinical cellular and in vivo models of brain ischemia and ICH [107,157,158], as well as in clinical studies of brain ischemia [159,160,161]. The neuroprotective effects promoted by H_2_ can be mediated also by a modulatory action on MCs, since the gas inhalation can reduce mastocytic activation through the inhibition of the FcεR-mediated signal transduction [109,162].

Carnosine (β-alanyl-l-histidine) is an endogenous dipeptide widely expressed in body tissues, including CNS [163]. Thanks to its antioxidant, pH-buffering, and metal ion-chelating properties, combined with good tolerability and safety profile, carnosine is commonly used as dietary supplement [163]. Carnosine and its derivatives have been reported to have a potential for the treatment of several pathologies, including brain ischemia [164,165,166,167,168,169]. Carnosine is also able to reduce MCs release of histamine [170]. Notably, carnosine attenuates mastocytic degranulation and histamine release induced by OGD [85], suggesting that the MCs stabilizer capability of the molecule can be involved in the beneficial effects observed in preclinical models of brain ischemia.

Emedastine difumarate and tranilast are two anti-allergic drugs endowed with inhibitory properties on MCs degranulation [171,172,173]. Treatment of an experimentally induced intracranial aneurysm rat model with emedastine or tranilast promoted a decrease in aneurysm size and an increase in thickness [119].

Mesenchymal stem cells (MSCs) are multipotent progenitor cells that can differentiate into several cell types in the adult tissues. For their anti-inflammatory effects, MSCs have been investigated for the treatment of inflammatory pathologies [174]. Intravenous injection of MSCs reduced aneurysm rupture rate and MCs infiltration in a intracranial aneurysm mouse model [175]. The modulatory effect of MSCs on MCs appeared to be mediated by the cyclooxygenase-2 (COX-2)-dependent production of prostaglandin E2 (PGE2) [175].

Masitinib mesylate is a multitargeted tyrosine kinase inhibitor studied for its potential antineoplastic activity [176]. Masitinib was also shown to reduce ischemic brain area and neurological deficits in a rat model of brain ischemia [177]. One of the mechanisms which could explain the neuroprotective effect of the molecule is its modulatory activity on MCs by inhibiting Lyn kinase [178].

Scopoletin (6-methoxy-7-hydeoxycoumarin) is a coumarin compound isolated from several plants [179]. The molecule, used in traditional Chinese medicine, have been investigated for its antioxidant and anti-inflammatory properties [179,180]. Recently, it has been reported that scopoletin is effective in mediating neuroprotection in a rat model of brain ischemia [181]. Moreover, scopoletin was shown to reduce the production of inflammatory cytokines in a human MC line [182]. Although a direct correlation between scopoletin and MCs modulation in stroke models is yet to be determined, it is plausible that reduction of MCs activation could play a role in the scopoletin protection against ischemic injury.

Resveratrol (trans-3, 5, 4′-trihydroxystilbene) is a natural polyphenol widely studied for its anti-aging, anti-inflammatory, antioxidant, and anticarcinogenic properties [183]. Resveratrol and its glucoside derivative polydatin showed beneficial effects in various cellular and animal models of stroke, either alone or in combination with other molecules [184,185,186,187,188,189,190,191,192,193]. When administered as an adjuvant with rtPA treatment, resveratrol showed to extend the clinical therapeutic window of rtPA, by improving the outcome of patients receiving late stroke treatment [194]. Resveratrol and polydatin can also modulate MCs functions by targeting IgE-dependent mastocytic activation [195,196,197,198,199,200,201]. In light of a possible correlation between IgE levels and stroke, these findings strongly suggest that MCs stabilization could play a role in the protection promoted by resveratrol and polydatin against ischemic damage.

Ketotifen is a second-generation antihistamine and MCs stabilizer used for the management of allergic disorders including asthma, allergic rhinitis/conjunctivitis, atopic dermatitis, and chronic urticaria [202,203,204,205,206]. Interestingly, ketotifen has been demonstrated to decrease the multiorgan damage and improve survival rate in rats subjected to intestinal ischemic reperfusion injury by inhibiting MCs activation [207]. Although the effect of ketotifen in stroke models has not been investigated yet, the molecule deserves future attention as potential approach to reduce neuroinflammation by targeting MCs. 

## 6. Conclusions

The studies summarized in this review indicate a detrimental role of MCs in various types of stroke. Through the release of their mediators, MCs can promote BBB damage, vasogenic edema, and hemorrhage formation, can recruit other immune cells amplifying inflammatory response, and can contribute to CA formation and vasospasm. 

In light of the very early role of MCs in stroke and their complex modulatory effect on other cell populations, pharmacological agents targeting MCs stabilization in the brain may offer an effective neuroprotective strategy for stroke, alone or in combination with current available therapies. Available randomized controlled clinical studies indicated that the treatments with some of the agents endowed with mastocyte modulatory activity, namely H_2_ and resveratrol, were safe and effective in stroke patients [148,159,160,161,194]. PEA/luteolin, investigated through an observational study in a cohort of 250 stroke patients, also showed a good tolerability and improved the outcome, when compared to literature data on patients having similar pathologic conditions but never receiving PEA/luteolin [148]. 

However, several challenges need to be addressed in order to modulate MCs activation in stroke. Immune responses after stroke, involving also MCs activation, are extremely complex, with many processes potentially having both beneficial and detrimental roles. Moreover, our understanding of the transcriptional and epigenetic dynamics driving MCs activation is still limited [37]. A better knowledge of the post-stroke mastocytic modulation, supported by mechanistic studies, will help to identify specific therapeutic targets and better approaches for stroke treatment. 

For example, many intracellular and extracellular mediators of MCs activation, cell-surface receptors involved in mastocytic modulation, and molecules released by activated MCs can be targeted by different molecules with potential beneficial effects in therapeutic intervention against stroke (for novel approaches in clinical targeting of MCs functions see [37,208]). Potential therapeutic targets in MCs include cytokines (such as TNFα, IL-1, IL-5, IL-9, IL-13, IL-17A, IL-33, GM-CSF (granulocyte-macrophage colony-stimulating factor)), IgE, histamine, MCs-surface receptors (such as IL-4Rα, cysteinyl leukotriene receptor 1 (CYS LTR1), β-2 adrenergic receptor (ADRB2)), arachidonate 5–lipoxygenase (ALOX5, involved in leukotriene synthesis), COX-2 (involved in prostaglandin synthesis), calcineurin (a phosphatase activating the transcription factor NFAT, involved in the expression of proinflammatory cytokines), the glucocorticoid receptor (inhibiting proinflammatory cytokine synthesis), and the inhibitory receptor Siglec 8 [37,208].

Finally, available data on MCs in stroke patients are limited [94,120,121,122,124]. Rodent and human MCs can vary in phenotype, responsiveness to activation signals, and the spectrum of preformed and newly synthetized mediators [6]. Therefore, findings on rodent MCs should be supported by studies on patients. Particular focus is required for the investigation of the role of meningeal MCs in stroke [92]. Identifying the location of the MCs population more involved in stroke pathology may foster the research of effective therapeutics. The development of a molecular imaging probe detecting MCs could help to address this issue.

## Figures and Tables

**Table 1:** Role of mast cells in the pathogenesis of stroke.

Type of Stroke	Experimental Model	Findings	References
**NHIBI**	Carotid ligation mouse model	MCs associated genes upregulated	[70]
	Carotid ligation rat model	Rapid increase of activated MCs in the brain	[71,72]
		MCs pharmacological inhibition reduced MCs migration, brain damage and glial activation	
	Transient focal ischemia rat model	Rapid increase of activated MCs and histamine in the brain	[73]
	Ibotenate mouse model	IL-9 exacerbated brain damage by activating MCs	[74]
		MCs pharmacological inhibition reduced brain damage	
**Ischemic Stroke**	OGD mouse MCs	OGD promoted MCs activation	[83,84,85]
	OGD mouse MCs and neurons	OGD-activated MCs induced neurotoxicity	[83]
		MCs pharmacological inhibition reduced MCs-induced neurotoxicity	
	MCAO mouse model	MCs associated gene upregulated	[87]
		MC-deficient mice showed decreased BBB leakage, brain edema and neutrophils infiltration	[90]
		MCs pharmacological inhibition decreased BBB leakage, brain edema and neutrophils infiltration	
		Meningeal MCs worsen infiltration of granulocytes and macrophages, brain swelling, and infarct size	[93]
	Four-vessel occlusion rat model	Modulation of MCs number and histamine levels	[88]
	MCAO rat model	MCs pharmacological activation increased edema formation	[89]
		MCs pharmacological inhibition decreased brain swelling, BBB leakage and neutrophils infiltration	
		MC-deficient rats showed decreased brain swelling, BBB leakage, and neutrophils infiltration	
	MCAO rat model	Increased MCs gelatinase activity	[91]
		MCs pharmacological activation increased gelatinase activity	
		MCs pharmacological inhibition decreased gelatinase activity	
		MC-deficient rats displayed decreased gelatinase activity	
	MCAO rat model treated with rtPA	MCs pharmacological inhibition reduced rtPA-induced hemorrhagic conversion, brain swelling, and neutrophil infiltration.	[98]
		MC-deficient rats displayed decreased rtPA-induced hemorrhagic conversion, brain swelling, and neutrophil infiltration.	
	Patients	Lack of MCs in penumbra brain region	[94]
**ICH**	Blood infusion rat model	MCs pharmacological activation increased brain damage.	[106]
		MCs pharmacological inhibition decreased brain damage, improved neurologic outcome	
		MC-deficient rats displayed decreased brain damage, improved neurologic outcome	
	Collagenase infusion mouse model	MCs activation	[107,108]
		MCs pharmacological inhibition decreased brain damage, improved neurologic outcome	
	Collagenase infusion rat model treated with rtPA	MCs pharmacological inhibition reduced rtPA-induced hematoma growth, hemispheric expansion, mortality, and neurologic deficits.	[109]
**SAH**	CA rat model	MCs in aneurysm wall	[119]
		MCs pharmacological inhibition reduced inflammation and CA size and thinning	
	Co-culture rat MCs and smooth muscle cells	Histamine and thromboxane inhibitors decreased MCs-mediated vasoconstriction	[119]
	Patients	MCs in aneurysm wall	[120,121,122]
		MCs in the muscular layer of cerebral arteries	[123]

BBB: blood brain barrier; CA: cerebral aneurysm; ICH: intracerebral hemorrhage; MCAO: middle cerebral artery occlusion; MCs: mast cells; NHIBI: neonatal hypoxic-ischemic brain injury; OGD: oxygen and glucose deprivation; rtPA: recombinant tissue plasminogen activator; SAH: subarachnoid hemorrhage.

**Table 2:** Therapeutic modulation of MCs in stroke models.

Drugs	Experimental Model	Findings	References
PEA	MCAO rat model	PEA reduced MCs derived chymase and tryptase	[137]
Luteolin	OGD mouse MCs and neurons	Luteolin reduced OGD-activated MCs degranulation and induced neurotoxicity	[83]
PEA/Luteolin	OGD mouse MCs and neurons	PEA/Luteolin reduced OGD-activated MCs degranulation and MCs-induced neurotoxicity	[83]
	MCAO rat model	PEA/Luteolin reduced ischemia-induced MCs infiltration and expression of chymase and tryptase	[148]
Cromoglycate	Carotid ligation rat model	Cromoglycate reduced MCs migration, glial activation and brain atrophy	[71,72]
	Ibotenate mouse model	Cromoglycate reduced MCs migration, glial activation and brain atrophy	[74]
	MCAO rat model	Cromoglycate reduced brain swelling, perivascular gelatinase activity, BBB leakage and neutrophil accumulation	[53,98]
	MCAO mouse model	Cromoglycate decreased BBB leakage, brain edema and neutrophils infiltration	[90]
	MCAO rat model treated with rtPA	Cromoglycate reduced rtPA-induced hemorrhagic conversion, brain swelling and neutrophil infiltration.	[98]
	Blood infusion rat model	Cromoglycate inhibited hematoma growth, decreased neurological deficits and mortality	[106]
	Collagenase infusion rat model treated with rtPA	Cromoglycate reduced rtPA-induced hematoma growth, hemispheric expansion, mortality and neurologic deficits.	[109]
IVIG	Collagenase infusion mouse model	IVIG attenuated BBB damage, brain edema, infarct area and pro-inflammatory cytokines levels	[108]
H_2_	Collagenase infusion mouse model	H_2_ decreased MCs accumulation and degranulation, BBB damage and improved neurobehavioral function	[107]
Carnosine	OGD rat MCs	Carnosine reduced degranulation and histamine release in OGD-activated MCs	[85]
Emedastine	CA rat model	Emedastine decreased MCs activation, inflammation and CA size and thinning.	[119]
Tranilast	CA rat model	Tranilast decreased MCs activation, inflammation and CA size and thinning.	[119]
MSCs	CA mouse model	Intravenous injection of MSCs reduced aneurysm rupture rate and CA MCs infiltration	[175]

Studies showing MCs modulation by masitinib, scopoletin, resveratrol, polydatin and ketotifen in stroke models were not available at the moment of the manuscript submission. BBB: blood brain barrier; CA: cerebral aneurysm; ICH: intracerebral hemorrhage; MCAO: middle cerebral artery occlusion; MCs: mast cells; MSCs: mesenchymal stem cells; NHIBI: neonatal hypoxic-ischemic brain injury; OGD: oxygen and glucose deprivation; rtPA: recombinant tissue plasminogen activator; SAH: subarachnoid hemorrhage.
